# PART and SNAP

**DOI:** 10.1007/s00401-014-1362-3

**Published:** 2014-11-08

**Authors:** Clifford R. Jack

**Affiliations:** Department of Radiology, Mayo Clinic and Foundation, 200 First Street SW, Rochester, MN 55905 USA

The condition described by Crary et al. [5] of predominantly medial temporal lobe tauopathy in the absence of β-amyloidosis has a clear parallel in the recent imaging/biomarker literature. Individuals with imaging/biomarker evidence of Alzheimer’s disease (AD)-like neurodegeneration without β-amyloidosis have been labeled “suspected non-Alzheimer’s pathophysiology (SNAP)” [8, 13, 18, 22, 27, 30, 31, 34, 37, 39, 41, 44].

Biomarkers of β-amyloidosis are amyloid PET and low CSF Aβ42. Biomarkers of AD-related neurodegeneration are high CSF tau (total or phosphorylated); atrophy on structural MRI in an AD-like topographic pattern (particularly medial temporal structures); and decreased metabolism on FDG-PET in an AD-like topographic pattern [14]. Positive or negative cut points for each biomarker modality have typically been established in relation to AD dementia subjects [8, 13, 18, 22, 27, 30, 31, 34, 37, 39, 41, 44]. By designating subjects as either β-amyloid positive or negative, and neurodegeneration positive or negative, every individual can be classified into one of four groups: neither amyloidosis nor neurodegeneration; amyloidosis without neurodegeneration; amyloidosis plus neurodegeneration; or, neurodegeneration without amyloidosis (i.e., SNAP). We [18] originally labeled this last group SNAP because we felt that neurodegeneration in this group represented non-AD etiologies; however, as discussed later in this commentary, the designation “non-AD” has been controversial. While the SNAP construct was initially described in cognitively normal elderly [18] it has also been applied to categorize mildly impaired individuals.

## Linking neurodegeneration in SNAP to PART pathology

The link between atrophy of medial temporal structures on MRI and the pathology of PART is straightforward, as is the link between abnormally elevated CSF tau and the pathology of PART. The link between decreased metabolism in AD-like areas on FDG-PET and the pathology of PART may, however, not be intuitively obvious. Medial parietal and lateral temporal/parietal cortical hypometabolism in PART could be explained by direct involvement of these areas by tauopathy which has extended beyond the medial temporal lobe as described in Crary et al. [5]. It could also be explained, however, by the fact that the medial temporal lobe (always involved in PART) is highly connected functionally to the posterior default mode network which is located anatomically in medial parietal and lateral temporal/parietal cortex and therefore overlaps extensively with the AD-like hypometabolism pattern [1, 2, 36].

## Parallels between PART and SNAP

At least 12 different studies in seven different cohorts have been published to date describing characteristics of SNAP in cognitively normal elderly subjects [13, 18, 20–23, 27, 34, 37, 39, 41, 44]. And at least three studies in four different cohorts have been published describing SNAP in MCI subjects [8, 30, 31]. Clear parallels exist between SNAP and PART in several areas.

First, while population frequencies of PART are not estimated in Crary et al. [5], PART is judged to be common in middle-aged and elderly subjects. SNAP is likewise common in subjects over age 65. Of 1,425 cognitively normal subjects reported from seven different centers, 315 (22 %) were categorized as SNAP [18, 22, 27, 37, 39, 41]. Of 277 MCI subjects reported from 4 different studies, 68 (25 %) were categorized as SNAP [8, 30, 31]. Given the large numbers of subjects included in these studies (esp. cognitively normal) the population frequency estimates of SNAP are likely reliable.

Second, APOE4 is underrepresented in both PART and SNAP. The frequency of APOE4 carriership among subjects with definite PART ranges from 9.1 to 20 % for different Braak stages (Table 1, in Crary et al. [5]). Among cognitively normal SNAP subjects, reported frequencies of APOE4 carriership ranged from 12 to 30 % [18, 22, 27, 41]. In all these studies, the frequency of APOE4 in SNAP was dramatically lower than in subjects with preclinical AD.

Third, the cognitive/clinical profile of both SNAP and PART is one of no impairment to mild cognitive impairment. Frank dementia appears to be rare in SNAP [24]. Mean MMSE scores among subjects with definite PART grouped by Braak stage (with average age in the 80 s) ranged from 28 to 24 Table 1 in Crary et al. [5]). SNAP in turn has been described in subjects who are either cognitively normal or MCI. Furthermore, longitudinal clinical follow-up of cognitively normal SNAP subjects reveals a somewhat benign trajectory where the risk of clinical/cognitive decline for SNAP is significantly less than subjects classified as both amyloidosis and neurodegeneration positive [22, 27, 34, 39, 41].

## Caveats concerning parallels between PART and SNAP

Drawing parallels between SNAP and PART comes with an important caveat—neurodegenerative imaging/biomarker non-specificity. The imaging findings used to define neurodegeneration in SNAP are not specific for temporal lobe tauopathy (i.e., PART). While hippocampal/medial temporal atrophy on MRI correlates well with tau burden and Braak stage [15, 43], other pathologies also produce hippocampal atrophy. These are well known to pathologists and include hippocampal sclerosis [15, 29, 33, 45], frontotemporal lobar degeneration (especially with TDP43 pathology [42]), argyrophilic grain disease, and ischemia/anoxia [7]. Temporal/parietal FDG-PET hypometabolism also occurs in conditions other than temporal lobe tauopathy, for example, cerebrovascular disease [44]. The same caveat applies to elevated CSF tau, which is seen in conditions other than PART including ischemic cerebrovascular disease, traumatic brain injury, and Creutzfeldt–Jakob disease [38].

These caveats notwithstanding, to date, autopsy results have been reported in 4 SNAP subjects [41]. Three of the four had low probability AD and the fourth was not AD by NIA–AA pathological criteria [12, 26]. Two of the four autopsy reports [41] described medial temporal tauopathy without amyloidosis—i.e., they met the definition of PART.

A solution for imaging/biomarker non-specificity may soon be at hand. Tau PET ligands have recently been developed [4, 25, 40] and the hope (or expectation) is that tau PET will reveal the contribution of tau to the neurodegenerative profile seen in subjects labeled SNAP on the basis of MRI, FDG-PET and CSF tau.

## The chief controversy: is PART a non-AD process or part of the AD spectrum?

As asserted in the accompanying commentary, the major controversy with PART is whether it should be considered an age-related non-AD entity or part of the AD pathological spectrum. Precisely, the same controversy exists in the imaging/biomarker literature on SNAP. Some in the imaging/biomarker community argue that evidence of β-amyloid deposition is required to label an individual as being in the “AD pathophysiological pathway” on the basis of biomarkers and hence SNAP is correctly labeled a non-AD condition. Others argue that because the neurodegenerative biomarkers in SNAP are AD-like, SNAP represents a “pre fibrillar amyloid” part of the AD spectrum [3]. Elegant arguments have appeared on both sides of this debate.

There may be a way, however, to reconcile these opposing viewpoints on both PART and SNAP. A series of recent publications in the imaging/biomarker literature have proposed the following step-wise scenario as a common pathological and biomarker sequence in late-onset AD [16, 17, 19, 28]. This proposed pathological sequence is in fact based on earlier autopsy literature [6, 9, 32].

1. Essentially everyone in the population develops PART at some point in life. Typically this occurs prior to significant fibrillar amyloid deposition. By itself, however, PART produces none to mild clinical symptomatology.
2. Independently from PART, β-amyloidosis develops in neocortical areas [10, 35].
3. At some point in time, which varies considerably from person to person and through as yet undetermined signaling mechanisms, β-amyloidosis begins to induce the spread of tauopathy from medial temporal to widespread neocortical association areas.
4. Severe clinical symptoms are due to direct involvement of neocortical areas by the accelerated and expanding tauopathy, not due to direct involvement by β-amyloid deposition.

In this model of late-onset AD [6, 9, 16, 17, 19, 28, 32], the role of β-amyloid is to induce the propagation of tauopathy, rather than to initiate the first tau deposition in the brain (as is likely the case in genetically determined AD [11]).

In summary, the paper by Crary et al. [5] formalizes a key concept that links autopsy findings to imaging/biomarker findings and fills a void that the imaging/biomarker community has struggled with for several years. By introducing the term PART and characterizing this entity Crary et al. [5] have provided the clinical, imaging/biomarker community with an important foundation on which to rest future studies.
